# Midwives’ views on the acceptability of a future trial of the Sims posture for fetal malposition in labor in the context of their knowledge and practice: A mixed-methods study

**DOI:** 10.18332/ejm/150377

**Published:** 2022-08-01

**Authors:** Jennifer Barrowclough, Caroline Crowther, Bridget Kool

**Affiliations:** 1 Liggins Institute, The University of Auckland, Auckland, New Zealand; 2 Section of Epidemiology and Biostatics, School of Population Health, Faculty of Medical and Health Sciences, University of Auckland, Auckland, New Zealand; 3 Midwifery department, School of Clinical Sciences, Faculty of Health and Environmental Sciences, Auckland University of Technology, Auckland, New Zealand

**Keywords:** midwives, fetal malposition, maternal posture

## Abstract

**INTRODUCTION:**

Evidence of safe and effective maternal interventions to improve fetal malposition in labor is inconclusive. A contemporary, randomized controlled trial of maternal posture would expand this evidence, however, collaboration with midwives will be critical. The aim of this study is to assess midwives’ views on the acceptability of a trial of the Sims posture for fetal malposition in labor and identify current midwifery knowledge and practice surrounding fetal malposition.

**METHODS:**

A mixed-methods study incorporating a web-based survey and guided focus groups with midwives was conducted in New Zealand during 2020. Midwives serving Auckland Hospital and Māori and Pasifika midwives serving South Auckland (n=136) were invited to participate in the study. Data were descriptively analyzed using chi-squared and cross-tabulation. Collaboration with a trial was contextualized by thematic content from survey and focus-group data.

**RESULTS:**

Fifty (36%) midwives from primary and secondary/tertiary settings responded to the survey, and 19 participated in four focus groups. Most midwives thought maternal posture affects malposition, utilize changes of posture often with the peanut ball, would recommend a posture if cesareans were reduced by 20%, and would definitely or probably collaborate with a labor trial of posture. Fetal monitoring with women in the Sims posture was difficult for nearly one-fifth of midwives. Seven themes emerged regarding trial participation: trial design, relevance, practice, diagnosis, knowledge and skills, and trial compliance.

**CONCLUSIONS:**

Current practice concerning malposition utilizes flexibility of posture. Provision of some free movement and reassurance surrounding trial equipoise may enhance trial collaboration.

## INTRODUCTION

Fetal malposition in labor that includes occiput-posterior (OP) and occiput-transverse (OT) positions has a prevalence of 15–33% in the first stage of labor and 8–21% at birth^1-3^. However, a more recent study of nulliparous women reported 52% were OP in the first stage, 44% OP/OT in the second stage and 14% OP/OT at birth^4^. Sonographic confirmation of malposition is preferable due to the inaccuracy of vaginal examination^1^. While approximately 80% of malposition resolves in labor^1,2^, it is associated with maternal morbidities including prolonged labor, use of epidural analgesia, augmentation of labor with oxytocin, operative vaginal birth, severe perineal trauma, cesarean section, and postpartum haemorrhage^3,5^. Infant morbidities associated with malposition include need for neonatal intensive care, birth injury including fracture, nerve palsy, head laceration, cephalohematoma, and longer hospital stay^6^.

The World Health Organization (WHO 1996) recommends non-supine positions in labor and freedom in position and movement throughout labor^7^. These guidelines may help prevent OP/OT presentation, however there is a lack of consensus on how to effectively manage malposition in labor^8,9^. The Royal College of Midwives notes the lack of evidence for interventions to correct fetal malposition in labor^10^. Postural interventions for fetal malposition include use of the hands and knees with or without pelvic rotation, tilting or shaking^8^, Walcher’s position^11^ and semi-prone or lateral postures with and without hip hyperflexion, which are the subject of a current Cochrane systematic review^12^.

Midwives are the main care providers during labor and birth providing effective maternity care globally^13^. Whilst legislation in New Zealand (Primary Maternity Services Notice 2021) provides women a choice of lead maternity carer (self-employed midwife, hospital-team midwife or private obstetrician); midwives are the predominant maternity care providers, providing around 87% of maternity care^14^. Any future randomized controlled trial (RCT) of posture for malposition in labor will require midwife collaboration, given that use of a single posture may conflict with midwives’ current practice guidelines.

This study aimed to assess the acceptability of a future RCT of maternal posture in labor for fetal malposition. Specific objectives were to assess: current midwifery practice for women with fetal malposition; the origins of the knowledge underpinning this practice; and enablers and barriers of a future RCT of maternal posture in labor for fetal malposition to improve maternal and infant health outcomes.

## METHODS

### Study design

A mixed-methods triangulation design based on a convergence parallel model was used^15^. In this method separate parallel quantitative and qualitative data analyses are merged to assess in what ways the findings converge and diverge. In this study, separate collections and analyses of survey and focus group data were performed simultaneously. The findings were converged during the interpretation to validate and enhance the quantitative survey data with the in-depth qualitative findings. An anonymous web-based survey created using Qualtrics software (Qualtrics, Provo, UT) comprised 19 questions including seven sociodemographic questions. Some display logic and open-ended questions were used. For example: ‘Do you have any views on what might cause fetal malposition? Please select one. Please explain what you think might cause fetal malposition’. Likert scales ranged 3–9. The survey was piloted by several midwives and refinements were made accordingly, for example inclusion of the words ‘please specify’ and use of a neutral facial expression for the illustrated figure. Focus groups with midwives were conducted by the midwife-researcher to contextualize and validate the survey findings. A semi-structured interview guide provided a framework for focus group discussions.

### Setting

Following ethical approval, invitations to participate in the survey and focus groups, including participant information sheets, were emailed via the staff newsletter by labor and birth managers at Auckland Hospital and Ngā Maia Aotearoa (A national Māori birthing association). Posters advertising the study were displayed on noticeboards within the birthing units. Midwives could access the survey via an email link or a QR code displayed on study posters. Paper copies of the survey were available on request. Completion of the survey was deemed to be informed consent. Survey completion time was about 7 minutes.

Written signed consent was collected from participants prior to focus group commencement. Focus group field notes were made by a non-midwife research assistant, independent of the study, who attended the focus groups and transcribed the audio recordings. A verbal summary of the discussion was given at the end of each focus group by the midwife-researcher to enable participants to confirm or refute whether the content of the session was accurately summarized. Transcripts from the focus groups were labelled FG1, FG2, FG3 and FG4, to maintain confidentially.

### Participants

Midwives providing labor and birthing care (n=126) during the past year at Auckland Hospital, (a tertiary maternity center with more than 6500 births annually) were invited to participate. Māori (New Zealand’s indigenous population) midwives are under-represented at Auckland Hospital^16^, therefore Māori midwives practicing in South Auckland (n=12), an area with a high proportion of Māori, were also invited to participate in the survey and focus groups. South Auckland Pasifika midwives frequently collaborate with Māori regarding under-representation issues; therefore, they were invited to participate in the focus group held in South Auckland.

### Measures

Measures were frequency, percentage and probability.

### Variables

Midwives’ knowledge, practice, and views on an RCT of maternal posture in labor for correction of fetal malposition were assessed including estimated number of overall labors and fetal malposition labors attended annually. Cultural views on the use of maternal posture may differ amongst New Zealand midwives and those of minority ethnicity may share or wish to represent the views of their community. Therefore, sociodemographic domains of interest included prioritized ethnicity (Stats NZ Level 1)^17^; midwifery practice (self-employed or employed); practice setting [home, primary hospital (no anesthetic/obstetric/neonatology services), or secondary/tertiary hospital]; highest midwifery qualification; additional education; and years of overall midwifery practice.

Midwives’ views on the causes of fetal malposition, related care practices, effects of maternal posture on fetal position, and sources of knowledge were sought. In addition, their views on the acceptability, safety and ease of fetal monitoring regarding the Sims posture^18^, described in the survey as ‘lying on the side inclined towards prone during labor’, and likelihood of participation in a trial of this posture were sought. Future trials will require consent from the pregnant woman; however, the term ‘participation’ was used in the survey as midwives’ collaboration was inherent.

Focus groups further explored their views on the acceptability of an RCT. Domains of interest included acceptability of participation in an RCT and perceived barriers and enablers for participation.

### Analyses

Survey quantitative data were described as frequencies and percentages. The probability of parametric data and non-parametric data were analyzed descriptively by cross-tabulation using chi-squared statistics in SPSS (SPSS for Windows version 27, SPSS Inc., Armonk, NY, USA). Fisher exact test was used where cells had values <5. Cross-tabulation was performed using chi-squared to determine the influence of respondent characteristics on participation in an RCT.

A general inductive approach was used to analyze the thematic content from the focus group and free-text survey data^19,20^. Trello software (Trello Inc., Atlassian) was used to organize and code free-text into themes and subthemes labelled by participant or focus group (e.g. P1 or FG1). Consensus regarding coding of themes was reached through discussion with a second reviewer (BK). Themes and subthemes were condensed into predominant themes/subthemes using a deductive approach, enabling less frequent yet important minor themes to remain. The qualitative data were reported according to the consolidated criteria for reporting qualitative research [COREQ] guidelines^21^.

## RESULTS

### Survey

The survey was available to access for five months during July–December 2020, extended beyond the planned two months due to the COVID-19 pandemic. During the survey period, 36% (n=50/138) of the eligible midwives completed the survey. The majority of respondents (n=37/50) accessed the survey via the link contained in the survey invitation email, one-quarter (n=7/50) accessed the survey via QR code, or completed printed surveys (n=6/50).

Over two-thirds of respondents were New Zealand European or European (n=33/50), four participants identified as Māori, nine as Pacific Peoples/Other, and four as Asian (Table 1). Over half (n=28/50) of the midwives were self-employed. Most midwives (n=44/50) provided labor care in a secondary or tertiary unit at least some of the time. Just under one-third of respondents (n=16/50) provided labor care in a primary birthing unit, and one-quarter provided labor care in client’s homes (n=13/50). Most had at least a Bachelor’s degree in midwifery (n=38/50) and 20 had engaged in additional tertiary education (including Certificate n=5, Diploma n=4, and ≥ Bachelor’s degree n=7). Over half of the midwives surveyed had >5 years’ experience (n=35/50), and 15 had >20 years’ experience. Most midwives (n=27/50) attended >50 women in labor, annually. Two-thirds (n=33/50) of midwives attended >10 women in labor with a fetal malposition, annually.

**Table 1:** Characteristics of midwifery survey respondents in Auckland, 2020 (N=50)

*Characteristics*	*n*
**Ethnicity**	
Māori	4
Pacific Peoples/Other	9
European	33
Asian	4
Type of current midwifery practice*	
Self-employed	28
Employed	27
Location of labor care*	
Primary unit	16
Secondary/tertiary unit	44
Home/other	12
**Highest midwifery qualification**	
Certificate	3
Diploma/Advanced diploma	9
Bachelor’s degree or higher	38
**Additional tertiary education/enrolment**	
Yes	20
No	30
**Years of practice**	
0–5	15
6–10	10
11–20	10
≥21	15
**Women in labor attended per year**	
1–10	6
11–30	9
31–50	8
≥51	27
**Women with fetal malposition attended per year**	
1–10	17
11–20	15
21–30	8
≥31	10

* Multiple answers

### Midwives’ knowledge and care practices for women with a fetal malposition

Respondents derived knowledge of fetal malposition from reflection on practice (n=41/50), discussion with peers (n=34/50), and ideologies (n=28/50). One-third (n=17/50) derived their knowledge from published research. Other sources of information included Chinese medical training, personal experience, and training workshops. Three-quarters of midwives (n=36/50) had views on what caused fetal malposition. Free text indicated these included maternal posture, pelvic and abdominal anatomy, being sedentary, lack of antenatal education, body mass index, and early epidurals. Thirteen midwives indicated they were either unsure or did not know what caused fetal malposition. Most (n=40/46) midwives agreed maternal posture affected fetal position.

**Figure 1:**
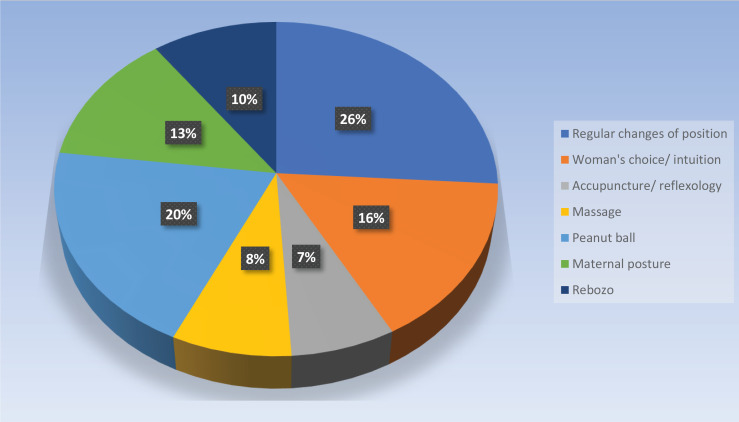
Care practices surveyed midwives use for women with fetal occiput posterior position in labor, in Auckland, 2020 (n=50)

Midwives commonly reported using a regular change of maternal position (n=42/50) and use of the peanut ball (an inflatable peanut shaped device placed between the knees) (n=33/50) as care practices for fetal malposition in labor (Figure 1). Other techniques selected from a range of 9 options included Rebozo technique (10%), women’s choice/intuition (16%), acupuncture/reflexology (7%), massage (8%), and maternal posture (13%). The range of maternal postures described by respondents in free text included hands and knees, side lying (including on the same side as fetal back), exaggerated Sims position, knees to chest, leaning forwards, Walcher’s position^11^, abdominal lift and tuck, correcting the pelvic tilt, position changes, pelvic rotation/shaking (including in a head down bottom up position), hip swing/press, kneeling, walking, walking sideways or stairs, standing, and upright sitting including on Swiss ball.

**Figure 2:**
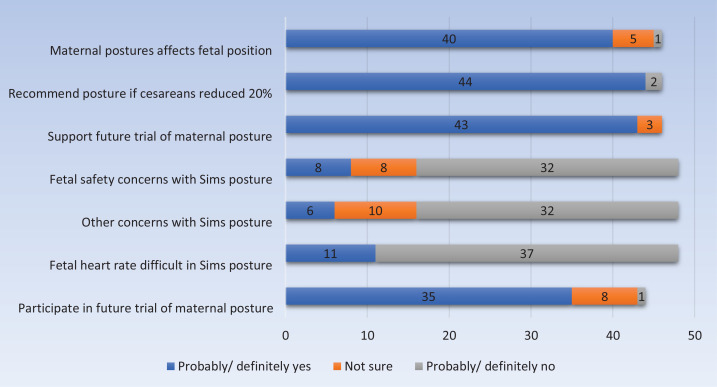
Midwife survey responses relating to the acceptability of a future trial of maternal posture for fetal malposition, in Auckland, 2020 (n=50)

Most midwives thought maternal posture ‘definitely’ or ‘probably’ affected fetal position in labor (n=40/50) (Figure 2) and most indicated they would ‘definitely’ (n=41/50) or ‘probably’ (n=3/50) recommend a specific posture to women if it ‘led to a 20% reduction in cesarean section births.

Most midwives (n=32/50) had no safety or other concerns regarding the Sims posture. Fetal monitoring in the Sims posture was viewed as definitely not or probably not difficult by nearly three-quarters of midwives (n=37/50) compared to ‘moderately or very difficult’ by just over one-fifth (n=11/50).

### Acceptability of a future trial and the influence of midwife characteristics

Midwives were ‘very supportive’ (n=31/50) or ‘mostly supportive’ (n=12/50) of a future trial of maternal posture, with no respondents opposed (Figure 2). Willingness to participate in such a trial was viewed as ‘extremely likely’ or ‘somewhat likely’ by over two-thirds of respondents (n=35/50). Participation was positively influenced by midwifery experience. Midwives attending 11–50 women in labor annually were ‘somewhat likely’ to participate (n=11/50) in a future trial, those attending >50 women in labor annually were ‘extremely likely’ to participate (n=14/50) whilst those who were [neutral] or ‘extremely unlikely’ attended only 1–10 women in labor annually (n=2/50) (p=0.014). Over one-third (n=18/50) of secondary or tertiary midwives were ‘extremely likely’ to participate compared to four midwives working only in primary locations who were ‘unsure’ (p=0.008).

### Focus groups

A qualitative study using four focus groups attended by 19 midwives was conducted during August and September in 2020. Participants attended venues at the tertiary hospital (n=9), a community maternity center (n=7) and a primary birthing unit (n=3). There was a mix of self-employed and employed midwives at the hospital focus groups, and the community focus groups were predominantly self-employed. A fifth of participants identified as Māori in discussion or attended the South Auckland birthing unit focus group and were therefore Māori or Pasifika. Focus groups ran for 30–45 minutes. All groups were satisfied that distributed verbal summaries accurately depicted discussions.

Initial analysis of transcripts produced 22 themes and 15 subthemes. Saturation of the themes^22^ was reached after FG3. FG4 provided additional nuances to common themes identified in earlier focus groups. Six dominant themes emerged with two to three subthemes per theme (Table 2). Links between themes were found through common subthemes. Themes included: practice, trial design, relevance of topic, knowledge and skills, diagnosis, and compliance.

**Table 2:** Midwife focus group themes and subthemes, in Auckland, 2020 (N=19)

*Theme*	*Subtheme*
**Practice**	Flexibility
	Toolbox
	Pride
**Trial design**	Fact finding
	Other trials
	Eligibility
	Incentives
	Control group inferior
**Relevance**	Cesareans
	Supportive of trial
	OP/OT version of normal
**Knowledge/skills**	Toolbox
**Diagnosis**	Accuracy
	Toolbox
**Compliance**	Comfort
	Intrusion
	Time
	If trusted
	Critical care

The ‘practice’ theme related to how midwives manage fetal malposition and included subthemes of flexibility, midwife’s toolbox, and pride. Focus groups held in primary settings expressed more optimism over their practice in cases of malposition. Midwives often reported using several postures in succession demonstrating a belief in flexibility of postures. Pride included pride of practice and fear of loss of pride. The ‘practice’ theme had links with ‘relevance of topic’ and ‘compliance’ (subtheme ‘supportive of trial’).

Examples of these subthemes are listed:

*‘It's usually posture so there is a sequence of different exercises you can do. You were with me on the course.’* (connectivity to the group) (FG1, PA)

*‘… don't we [normally have women], not in one position?’* (flexibility) (FG2, PB )

*‘It would be really good to give midwives another kind of tool in their kete [bag].’* (tool-box) (FG4, P2)

*‘I've seen … core staff taking real pride in it [the peanut ball].’* (pride) (FG1, PR)

*‘… would feel a lot more comfortable without the judgment either, midwives could do it [preferring an observational study].’* (pride) (FG4, P2)

‘Trial design’ was a broad theme encompassing some participants questioning whether a trial was necessary, whilst others made suggestions regarding the trial design. Subthemes included fact finding, other trials, eligibility, incentives, and control group inferior. Some interpreted the control group as control of practice rather than control of the experiment, so it was reiterated it meant usual care. There was consensus that incentives for women were expected but mixed opinion regarding incentives for midwives. A before and after observational study was preferred by some midwives to reduce conflict regarding trial allocation if the intervention was perceived preferable. ‘Trial design’ inter-linked with the themes practice (subtheme flexibility), diagnosis (subtheme accuracy) and compliance (subtheme comfort). Quotation examples include:

*‘What is care as usual?’* (fact finding) (FG2, PZ)

*‘[In another] study through labor … we didn't get put into control groups, we just did a survey after’ and ‘… it's a shame that it couldn't be like a retrospective study on how we [currently] practice without restricting or prescribing positions.’* (other trials) (FG4, P2)

*‘… if you don't [include …] then you're taking out quite a lot of women that have inductions … [who] are most likely to end up with malpositioned babies because they're on their backs …’* (eligibility) (FG1, PR)

*‘… if we want to support our families … [then] we don't need payment.’* (incentives) (FG3, P1)

*‘I'd find it really hard … in the control group and the posture was in the experimental, not to do that technique.’* (control group inferior) (FG1, PR)

‘Relevance’ described how relevant research into fetal malposition in labor was perceived. The theme was widely viewed as important due to the prevalence of malposition and associated operative births. Subthemes were ‘cesareans’ and ‘supportive of trial’. However, one participant described the OP/OT position as a variation of normal because fetal position can change. The theme thus had links with ‘practice’ and ‘compliance’ (subtheme ‘supportive of trial’). Examples of these subthemes include:

*‘I think the majority of cesarean sections that actually happen in the hospital is very likely to do with malposition or malpresentation and failure to progress.’* (cesareans) (FG1, PA)

*‘… the concerning operative rates … posterior babies are hugely alarming for me …’* (cesareans) (FG4, P3)

*‘Yeah, I think it's great.’* (supportive of a trial) (FG3, P4)

*‘Just because we see it a lot … Big babies, with epidurals. It happens a lot here.’* (supportive of a trial) (FG3, P3)

*‘… yeah it's harder, but it's actually another version of normal … That doesn't mean it's not able to be birthed vaginally.’* (OP/OT a version of normal) (FG4, P3)

The theme ‘knowledge and skills’ conveyed a high level of support for a research trial through development of knowledge and tools to improve outcomes. This theme linked with the theme ‘practice’, for example:

*‘It would be good to have some more tricks up the sleeve.’* (FG3, P4)

*‘… a research trial would … back up what we're already trying to say about women's positioning in labor.’* (FG4, P2)

‘Diagnosis’ relates to how fetal malposition is determined. There was consensus that determining fetal malposition by vaginal examination is sometimes difficult. Subthemes were ‘accuracy’ and ‘toolbox’ through the acquisition of sonographic skills. Other skills including abdominal palpation were proffered as diagnostic tools. For example:

*‘… most people do find it hard … they're like, ‘it's really hard to tell’… so … how accurate is it going to be.’* (accuracy) (FG3, P3)

*‘…There are signs, there is delayed second stages. So many signs that you can put together that you know the baby is OP.’* (accuracy) (FG1, PA)

*‘Yeah, scanning a new skill.’* (tool-box) (FG2, P4)

*‘I would love to learn how to scan … Yeah, I'd like to … it'd be great.’* (tool-box) (FG1, P3)

‘Compliance’ describes maternal and midwifery compliance to the protocol. The former frequently related to the woman’s comfort or restlessness, however inclusion of epidurals in the protocol brought reassurance. Subthemes included ‘comfort’, ‘intrusion of research’, ‘if trusted’, ‘critical care’ for example if responses to fetal bradycardia resulted in a breach of protocol, and ‘time constraints’ for paperwork and compliance checks for which tick boxes were preferred. This theme linked with ‘trial design’ (subtheme ‘control group inferior’) and ‘practice’. Examples of the subthemes include:

*‘Is she able to stay in that one position for so long?... With the epidural that would be ok.’* (comfort) (FG2, PB)

*‘… don't know if I like the idea of someone extra because we have people come in all the time already.’* (intrusion) (FG2, PK)

*‘I don't know that we've got time to be checking on one another. But … if we've committed … we'll do it.’* (time) (FG2, PJ)

*‘Why would you need to countersign? Can you not just like, trust the practitioner?’* (if trusted) (FG3, P4)

*‘… for the fetal heart.’* (critical care) (FG2, P1)

*‘... epidurals that are more effective on one side than the other.’* (critical care) (FG2, P2)

## DISCUSSION

This study assessed the knowledge and practices surrounding fetal malposition in labor of Auckland midwives who participated in surveys and focus groups, and their attitudes toward a future trial of maternal posture for correcting fetal malposition in labor. The ethnicity of the survey respondents closely resembled the hospital midwifery staff profile^16^ complimented by self-employed midwives. Inviting South Auckland Māori midwives to participate was an important step to reflect Māori.

### Strengths and limitations

Strengths of the study include: rarely collected information on midwives’ views surrounding malposition, rich ethnographic context of enablers and barriers for a future trial protocol, and insight into midwives’ attitudes that may impact on trial recruitment^23^. The findings reflect a range of Auckland midwives by location of practice, type of midwife, and ethnicity, enabling the findings to be generalized. The midwife-researcher brought inside knowledge of the labor care milieu to build the framework for discussion. The nature of a homophilous relationship may have aided recruitment^24^, though given the high commitment required to attend a focus group this effect may have been limited.

The findings need to be considered in the light of several limitations. The survey may have yielded different results had a higher response rate been achieved. The burden of restricted practice and stress during the COVID-19 pandemic is likely to have negatively affected response rates. Whilst potential respondents were followed up, incentives were not offered, which are known to increase participation^25^, due to the challenge of distribution via an anonymous online survey. The survey topic of maternal posture for fetal malposition, could have biased how midwives responded to questions concerning knowledge and practice of fetal malposition. The potential risk of researcher bias on interpretation of qualitative data was mitigated by using a second non-midwife reviewer to agree on coding.

The 36% survey response rate from midwives was lower than in other studies, including a meta-analysis of 48 surveys of nurses, physicians and allied health professionals, mostly from the United States (53%)^25^, and two surveys in Australia of midwives (57%)^26^ and obstetricians^27^ concerning their views on manual rotation. The rate of saturation of themes was consistent with a thematic analysis of 40 focus groups with healthcare consumers^22^.

The ethnicity of midwives in the survey closely resembled the hospital midwifery staff profile^16^ complimented by self-employed midwives.

### Knowledge and practice

Over 80% of midwives thought maternal posture affects fetal position and had views on what causes malposition. None of the respondents referred to the effect of gravity on fetal position, despite gravity underlying the hypotheses of several published trials^8,28,29^. Furthermore, only one-third of midwives derived knowledge of malposition from published research. Whether this reflects the lack of consensus in published literature on best practice for malposition or reflects a gap in knowledge translation^30^ is uncertain. Midwives' current practice broadly reflects the hospital guideline ‘Intrapartum Care - Physiological Labor and Birth’^31^, which cites the WHO^7^ recommendation to encourage use of non-supine positions and freedom in position and movement throughout labor. Use of the peanut ball was a popular practice. An RCT reported use of the peanut ball by primiparous women in first stage labor did not shorten the duration of labor or reduce fetal malposition or cesarean sections, however, it did provide comfort^32^.

### Acceptability of a future RCT

The majority of midwives indicated they would recommend a specific maternal posture for correction of a fetal malposition if it reduced the risk of a cesarean section by 20%. A similar readiness to change practice was reported in surveys assessing midwives and obstetricians’ readiness to perform manual rotation in the second stage of labor to reduce cesareans by 15–18%^26,27^. Midwives were widely supportive of future labor research on fetal malposition and were either unsure or would participate in trials of maternal posture for malposition. Training workshops on fetal monitoring in different maternal postures may build on midwives’ confidence given nearly one-quarter considered this difficult in the Sims posture.

A future RCT was viewed positively by most midwives, including midwives who wanted evidence for postures already used in practice. Some differences were found between focus groups. For example, focus groups held in primary care settings expressed more optimism regarding their current practice in cases of malposition, which may reflect the autonomous and continuous model of care within self-employed midwifery. However, this optimism may relate to the 80% of fetuses that do rotate anteriorly from a malposition in labor^1,2,33^ rather than the fetuses that remain persistently OP/OT. The secondary/tertiary setting focus groups were more pessimistic of expected outcomes of malposition in labor, which may reflect the outcomes and tertiary response to the 20% of women with persistent malposition.

### Enablers for future trial collaboration

Accurate diagnosis of malposition through the acquisition of sonographic skills was popular with midwives consistent with another study in which three-quarters of midwives indicated a desire to acquire the skill^26^. Considering sonographic diagnosis of malposition by a novice is accurate following brief training^34^; acquisition of this skill by midwives could eliminate the intrusion of qualified sonographers during labor whilst enabling blinding of the participating midwife. Focus groups endorsed the use of incentives for pregnant trial participants reflecting the Cochrane Review findings of Houghton et al.^23^. The use of tick boxes for any data collection required by the midwife would enhance collaboration as it had proven quick and easy in other trials. The majority of survey respondents considered the Sims posture safe, which may reflect a recent local study finding improved fetal oxygenation during lateral maternal postures compared to supine postures^35,36^.

### Barriers for future trial collaboration

The tendency to manage malposition according to normal labor guidelines which encourage flexibility of postures and mobility, may present a challenge for collaboration with a trial of a single posture especially if midwives anticipate discomfort from extended time in the posture. Interestingly, use of a single specified posture was assessed as acceptable by women participating in an RCT of hands and knees posture for fetal malposition in labor^29^. Furthermore, if a specific posture was found to facilitate anterior rotation, women may regard a degree of restriction as worthwhile. Other barriers related to a perceived lack of equipoise between the trial groups if the intervention posture was unavailable to the control group or the intervention group lacked the flexibility of the control group. It is possible some midwives misunderstood the word ‘control’ to mean restriction. On occasion, fast evolving conversation during the focus groups prevented clarification of terminology used by participants. The terms ‘free posture’ and ‘intervention posture’ rather than experimental and control groups, may improve comprehension of pre-trial information. Whilst midwives’ pride of practice was a minor theme, feeling judged on outcomes of midwifery care may present a psychological barrier for midwives to collaborate with an RCT.

## CONCLUSIONS

Our study suggests current midwifery practice concerning fetal malposition utilizes flexibility of maternal posture. Whilst midwives were supportive of a future RCT of maternal posture for fetal malposition, midwifery collaboration may be enhanced if the intervention includes periods of mobility, and training is provided for fetal monitoring in the Sims posture. Furthermore, staff preparation with an emphasis that no intervention is superior until rigorously tested, may address any concerns surrounding trial equipoise and eliminate conflict over trial group allocation.

## Data Availability

The data supporting this research are available from the authors on reasonable request.
